# Does using the sociodental approach in oral health care influence use of dental services and oral health of adolescents living in deprived communities? a one-year follow up study

**DOI:** 10.1186/s12913-023-09596-0

**Published:** 2023-06-09

**Authors:** Andressa Coelho Gomes, Mario Vianna Vettore, Larissa Neves Quadros, Maria Augusta Bessa Rebelo, Janete Maria Rebelo Vieira

**Affiliations:** 1 School of Dentistry, Federal University of Amazonas, Av. Ministro Waldemar Pedrosa, 1539, Praça 14 de Janeiro, Manaus, AM CEP 69025-050 Brazil; 2 Department of Health and Nursing Sciences, Faculty of Health and Sports Sciences, University of Agder, Campus Kristiansand, Universitetsveien 25, Kristiansand, 4630 Norway

**Keywords:** Oral health, Needs assessment, Longitudinal studies, Structural equation modelling

## Abstract

**Background:**

Oral health needs assessment is important for oral health care planning. This study compared dental treatment needs between normative and sociodental needs. We also longitudinally examined the relationships of baseline sociodental needs measures and socioeconomic status with one-year follow up measures of use of dental services, dental caries, filled teeth, and oral health-related quality of life (OHRQoL).

**Methods:**

A prospective study was conducted with 12-year-old adolescents from public schools in deprived communities in the city of Manaus, Brazil. Validated questionnaires were used to collect adolescents’ sex and socioeconomic status, OHRQoL (CPQ_11 − 14_) and behaviours (sugar intake, frequency of toothbrushing, regular use of fluoridated toothpaste and pattern of dental attendance). Normative need was assessed according to decayed teeth, clinical consequences of untreated dental caries, malocclusion, dental trauma, and dental calculus. The relationships between variables were tested thorough Structural equation modelling.

**Results:**

Overall 95.5% of adolescents had normative dental treatment needs. Of these, 9.4% were classified as high level of propensity. Higher normative/impact need and greater propensity-related need directly predicted use of dental services at one-year follow up. The latter mediated the association of normative/impact need and propensity-related need with incidence of dental caries and filled teeth. Normative/impact need and use of dental services were directly associated with filled teeth at one-year follow up. Poor OHRQoL at one-year follow-up was directly predicted by higher normative/impact need at baseline and less filled teeth at one-year follow up. Greater socioeconomic status was directly associated with better propensity-related need. Socioeconomic status indirectly predicted incidence of dental caries and filled teeth via propensity-related need and use of dental services.

**Conclusions:**

Sociodental needs measures were related to use of dental services, dental caries, filled teeth and OHRQoL after one year among adolescents living in deprived communities. Adolescents with dental needs treatment priorities according to the sociodental approach had more filled teeth via use of dental services. Dental services utilisation did not attenuate the impact of normative and impact-related need on dental caries incidence and poor OHRQoL after one year. Our findings suggest the importance of developing oral health promotion and enhancing access to dental care to improve oral health of adolescents living in deprived communities.

**Supplementary Information:**

The online version contains supplementary material available at 10.1186/s12913-023-09596-0.

## Background

The assessment of population oral health needs represents the basis for oral health care planning through providing relevant information for costs estimation, rational allocation of healthcare services and dental treatment expenses [1, 2]. Despite the improvements in populations’ oral health in several countries, oral health inequalities persist as an important public health problem [3]. The burden of oral diseases has been increasing in most parts of the world, and the treatment of oral diseases is extremely costly for families and health systems [4]. The estimated worldwide expenses due to oral diseases in 2015 totaled US$ 544.41 billion, including direct costs (treatments) and indirect costs (loss of productivity) [5]. In developing countries, the high costs of oral health care combined with insufficient health budget indicate an even worse scenario [4], resulting in a serious gap between population treatment needs and the available resources [6].

The common assumption in the organization and provision of dental care that the need for treatment should be normatively determined by dentists has been criticized. Essentially, oral health needs assessment should extend beyond the narrow and limited clinical interpretation of dental conditions. Other relevant factors, such as subjective perception of oral health and dental needs, individual behaviours, impact of oral health conditions on daily life and well-being, and social inequalities, should also be considered in dental care planning [1]. In addition, most normative methods for assessing oral health needs are considered unrealistic for dental services since they tend to overestimate the needs of the workforce and resources [2, 7].

In this context, the sociodental approach was developed as a new conceptual model to improve the current approaches of oral health needs assessment at population level [1, 2, 8]. The sociodental approach is a comprehensive method that integrates the measurement of the impact of oral conditions on quality of life and propensity-related measures to adopt health-related behaviours with normative assessment of oral health status [1, 2]. According to the sociodental framework, sociodental approach for the assessment of needs of oral health care is composed of the three following elements [1, 2, 8]. (1) Normative need (NN) evaluated through dental clinical measures. (2) Impact-related need (IRN) combined NN with an oral health-related quality of life (OHRQoL) measure. IRN is applied only for non-progressive oral conditions that are unlikely to progress and are not life threatening, such as malocclusion, enamel trauma, missing teeth, and periodontal diseases. (3) Propensity-related need is estimated by integrating IRN with propensity to adopt behaviours that may influence oral health and dental treatment outcomes. Dental treatment is recommended considering the likelihood of success, using the best available evidence about the effectiveness of treatments and the individual behavioural propensity. Individuals are categorized as high, medium, and low-propensity need. High propensity group includes those with good behavioural propensity who will most benefit from treatment, whereas those in the low-propensity group are at high risk of treatment failure. The latter group should initially receive intermediate or palliative treatments. In addition to clinical intervention, these individuals should receive oral health education and/or health promotion programs [2]. Two models of oral health care needs were outlined within the sociodental approach. The dental needs for life threatening and progressive oral conditions (DNLP) and the basic model for dental needs (BMDN). The former involves dental conditions with a high probability of progression or requiring emergency treatment, including dental caries, precancerous lesions, and trauma involving dentin/pulp. IRN is not relevant and not evaluated for DNLP since these conditions need treatment regardless of their oral impacts [1, 2, 8].

The main purpose of the sociodental needs models is to overcome the limitations of using only normative measures for planning the provision of oral health care services through identifying and prioritizing those individuals who would most benefit from dental treatment [1, 2]. Previous studies demonstrated that dental needs using the sociodental approach were significantly lower than the normative need method when different oral conditions, such as dental caries, gingivitis, periodontal disease, dental trauma, malocclusion, and need for dental prosthetics were assessed in children and adults [6–11]. These findings suggest that subject-centred sociodental needs assessment may provide a more realistic measurement of need since it considers the impact of oral conditions on quality of life and the propensity to adopt health promoting behaviours.

To date, the sociodental needs assessment was predominantly tested through cross-sectional studies as a framework for assessing dental needs. Thus, it remains unclear whether combining normative need, impact-related need and propensity-related need would influence use of dental services, dental clinical measures and OHRQoL over time. Longitudinal studies testing the sociodental approach of dental treatment needs can contribute to enhance the understanding of its application and utility in public oral health care services. Therefore, the aims of the present study are to compare the dental treatment needs between normative and sociodental dental needs models among 12-year-old adolescents living in deprived communities. Moreover, this study also evaluate the relationships of baseline sociodental needs measures and socioeconomic status with one-year follow up measures of use of dental services, dental caries, filled teeth, and OHRQoL. It was hypothesized a priori that adolescents with greater sociodental needs and higher propensity-needs at baseline are more likely to use dental services, have lower incidence of dental caries, more filled teeth and poor OHRQoL after one year of follow-up.

## Methods

### Study design and eligibility criteria

A one-year prospective longitudinal study was carried out in deprived communities in the eastern zone of the city of Manaus, Brazil, which has the poorest social indicators of the city (Gini index equal to 0.440). Inclusion criteria were adolescents enrolled in one of the selected public schools and age equal to 12 years. Exclusion criteria were adolescents in need of special care, with diagnosis of any syndrome and those using orthodontic appliances.

### Sampling process and power

A representative sample of 12-year-old adolescents enrolled in the 7th grade of municipal public schools was selected through stratified random sampling according to the size of the school population in the 11 districts of the city. Initially, twenty-five schools were randomly selected, proportional to the number of schools per neighbourhood. Thereafter, all students aged 12 years in the 7th grade from all classes of the selected schools were invited to participate. The multilevel structure of the data was assessed through testing the variance and standard error of null multi-level models considering school as a second-level variable. The variation of use of dental services at one-year follow up (P = 0.335), dental caries incidence at one-year follow up (P = 0.999), filled teeth incidence at one-year follow up (P = 0.712), and OHRQoL at one-year follow up (P = 0.575) were not statistically significant. Therefore, multilevel analysis accounting for school-level was not conducted.

Initially, 528 adolescents were invited to participate. Of them, 86 did not return the consent form or their parents did not agree with their participation, resulting in a sample 442 adolescents (baseline response rate = 76%). Twenty-seven adolescents were excluded due to the use of orthodontic appliances. Thus, the sample size at baseline included 415 adolescents. One-year follow-up data collection involved 334 adolescents, respectively (retention rate = 80.5%). A study with 334 participants would lend a power of 90% to estimate a structural equation model involving 5 observed variables and 2 latent variables considering a significance level of 5% and significant effects of 0.19 [12].

### Data collection

Adolescents completed self-administered questionnaires in 2016 to collect baseline data on sex, OHRQoL and oral health-related behaviours, including sugar intake, frequency of toothbrushing, and regular use of fluoridated toothpaste. Baseline information on socioeconomic status and pattern of dental attendance were collected from their parents/guardians using structured questionnaires.

Clinical oral examinations were performed by nine trained and calibrated dentists, using dental plain mirror (Duflex ®) and a ball-end community periodontal index probe (Stainless ®) under natural light in a sitting position. All adolescents performed oral hygiene under supervision before dental examinations. Number of decayed teeth, number of filled teeth, clinical consequences of untreated dental caries, malocclusion, dental trauma, and dental calculus were registered at baseline. Use of dental services, OHRQoL, dental caries and filled teeth were also collected at one-year follow up. The data was collected in a private room in the schools.

### Baseline measures

#### Sociodental need assessment

In this study, sociodental needs assessment was composed of NN, IRN and propensity-related need to test the DNLP and BMD models. Decayed teeth and clinical consequences of untreated dental caries were used in the DNLP model. Number of decayed permanent teeth and number of teeth with clinical consequences of untreated dental caries were evaluated using the decayed component of the Decayed, Missing and Filled Teeth Index (DMFT) [13] and the pulpal involvement (P/p), ulceration caused by dislocated tooth fragments (U/u), fistula (F/f) and abscess (A/a) (PUFA/pufa) index [14], respectively.

The BMDN applies to dental conditions that are less likely to progress or cause adverse health consequences in the absence of treatment [2]. Malocclusion, dental trauma without dentin/pulp involvement, and dental calculus were used to assess NN of BMDN in the present study. Malocclusion was assessed using the Dental Aesthetics Index (DAI) [15]. Adolescents with malocclusion were those with DAI score ≥ 26, including those in the following categories: definite malocclusion, severe malocclusion, and very severe or handicapping malocclusion [16]. Dental trauma without dentin/pulp involvement was assessed using a modified version of the O’Brien trauma index [17]. Dental calculus was assessed according to the modified Community Periodontal Index (CPI). Each tooth in an upper quadrant randomly selected using a randomization table and the contralateral lower quadrant were examined to register the presence or absence of dental calculus. The participants were considered with NN if at least one of the following dental clinical measures was registered: ≥ 1 decayed tooth, ≥ 1 tooth with clinical consequences of untreated dental caries, malocclusion (DAI score ≥ 26), ≥ 1 tooth with dental trauma, and/or ≥ 1 tooth with dental calculus.

Impact-related need was assessed using the validated version of the Child Perceptions Questionnaire (CPQ_11 − 14_) impact short form for Brazilian population [18]. CPQ_11 − 14_ consists of 16 items grouped into four dimensions: oral symptoms, functional limitation, emotional state, and social well-being. Each item is assessed using a four-point Likert scale with following response options: 0 = never, 1 = once or twice, 2 = sometimes, 3 = often, 4 = every day or almost every day. The total score is obtained by summing all items, which may range from 0 to 64. The higher the CPQ_11 − 14_ the greater the impact of oral health on quality of life. Impact-related need was registered for participants who responded ‘often’ or ‘every day or almost every day’ in at least one CPQ_11 − 14_ item.

Propensity-related need was assessed based on four oral health-related behaviours: frequency of daily sugar intake (0–3, 4–5, 6 or more times a day) [19], frequency of tooth brushing (twice or more times a day, once a day, not every day), use of fluoridated toothpaste (yes, no) and pattern of dental attendance (last dental visit within the last 12 months, last dental visit between two and three years, last dental visit more than three years ago) [2, 8]. Adolescents with a frequency of sugary foods/drinks from 0 to 3 times a day, frequency of tooth brushing twice or more times a day, use of fluoridated toothpaste and at least one dental visit in the last 12 months were classified as having a high behavioural propensity. Adolescents with medium behavioural propensity were those who answered at least one item at a moderate level and none at a poor level. Adolescents were classified as having a low propensity if at least one item was answered at a poor level [2, 8].

Sociodental dental needs assessment is a sequential integrated process for three levels of treatment needs measurement combining NN, IRN, and propensity-related needs supported by theoretical framework [1, 2, 8]. The first level is NN assessment according to DNLP and BMDN models. Then, IRN is assessed by integrating NN with subjective perceptions assessment using an OHRQoL measure in the second level. Individuals with NN and impacts of oral health on quality of life have IRN and should be prioritized. Those with NN and no impacts of oral conditions on quality of life should receive dental health education with the aim to improve their health behaviours. They may follow the treatment pathway and follow the propensity-related needs based on individual’s behavioural propensity according to the third level of measurement. People are categorized into high, medium and low levels of propensity-related needs. Then, available dental treatments and oral health care may be available for the different groups [1].

#### Socioeconomic status

Socioeconomic status was measured according to house crowding, number of goods in the household and monthly family income. House crowding was computed by dividing the number of people living in the household by the number of rooms in the house. Number of goods was measured according to the presence of eleven durable goods in the household. Monthly family income was the sum of all kinds of earnings (e.g. wages, pensions) of all family members living in the household in the last month. Income was registered in Brazilian minimum wages (BMWs) in 2016 using the following categories: 1 = up to half BMW, 2 = between half BMW and one BMW, 3 = more than one BMW. One BMW corresponded to R$ 881.00 Brazilian reais (U$ 271.00) in 2016.

#### Follow-up data collection

Adolescents were invited to participate in the follow-up assessment one year after baseline data collection. They were asked to inform whether they have visited a dentist for any reason during the last year and complete the CPQ_11 − 14_ questionnaire. Furthermore, they were re-examined for dental caries and number of filled teeth in the schools using the same dental exam protocol used in the initial examination. Dental caries incidence was evaluated according to the number of new decayed teeth, which was computed according to the number of ‘healthy teeth’, ‘filled teeth without caries’, and ‘teeth with sealant’ at baseline and coded as ‘decayed’ and ‘filled teeth with caries’ at one-year follow up. The difference on the number of ‘filled teeth without caries’ between one-year follow up and baseline was used to measure the incidence of filled teeth.

The participants’ schools and classes registered at baseline were used to reach them at one-year follow up. Those who were not found in their classes after at least three attempts were contacted using the telephone number obtained at baseline interview.

### Theoretical model

**Fig. 1:**
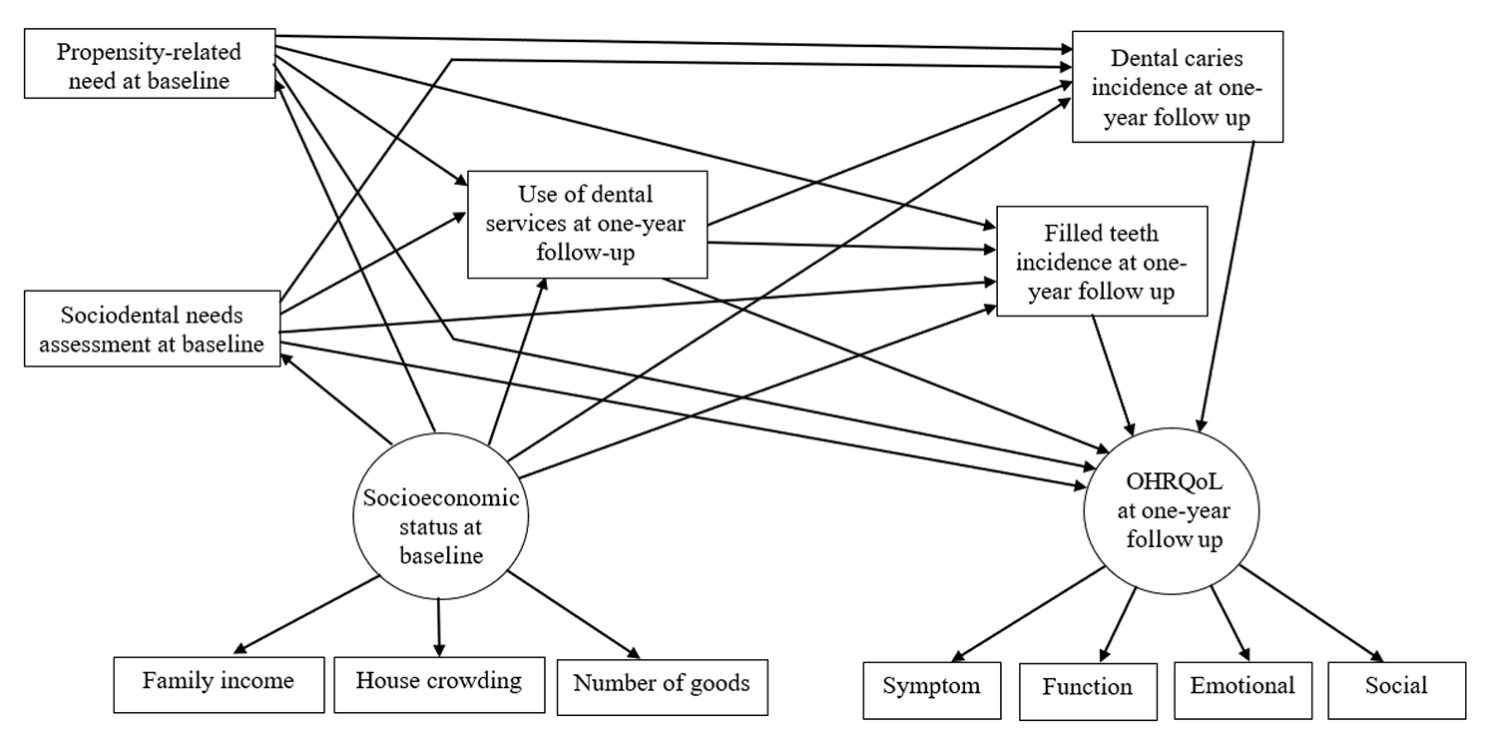
Hypothesized theoretical model on the relationships between sociodental needs measures, socioeconomic status, use of dental services, dental caries, filled teeth, and oral health-related quality of life

The conceptual framework of this study was based on the components of the sociodental system of needs assessment for dental needs and socioeconomic inequalities and their potential influence on use of dental services and oral health outcomes [1, 2, 8] (Fig. 1). According to the theoretical model, higher sociodental needs assessment combining normative need and impact-related need at baseline, and greater propensity-related need at baseline were expected to directly predict higher use of dental services, lower dental caries incidence, higher filled teeth incidence and worse OHRQoL at one-year follow-up. It was also hypothesized that better socioeconomic status at baseline, assessed through house crowding, number of goods in the household and monthly family income, would predict lower sociodental needs at baseline, better propensity-related needs at baseline, higher use of dental services, lower dental caries incidence and higher incidence of filled teeth at one-year follow-up. In addition, it was expected that use of dental services after one-year follow up would mediate the relationships of sociodental needs and propensity-related at baseline with dental caries incidence, filled teeth incidence and OHRQoL at one-year follow-up.

### Pilot study and clinical calibration

A pilot study was conducted involving ten adolescents who did not participate in the main study. Examiners were four dentists at baseline and five dentists at one-year follow up. Inter- and intra-examiner calibration for dental clinical examination tested the reliability of DMFT index, PUFA/pufa and DAI by performing two examinations on each adolescent within a one-week interval. The adolescents also completed the questionnaires to verify the understanding of the items used to evaluate health-related behaviours and OHRQoL. During the main study, 10% of participants were randomly re-examined to assess the reliability of the data. The reproducibility of instruments and questionnaires was evaluated using kappa coefficient for categorical variables and Intraclass Correlation Coefficient (ICC) for continuous variables.

The inter-examiner kappa coefficients for DMFT and PUFA/pufa at baseline were 0.951 and 0.730, respectively, and 0.796 and 0.863 at one-year follow up, respectively. Intra-examiner kappa coefficients for DMFT and PUFA/pufa ranged from 0.805 to 0.753 at baseline and 0.905 and 0.832 at one-year follow up, respectively. Inter-examiner agreement of DAI was performed using the consensus of two specialists in orthodontics with previous experience in oral health surveys as the gold standard. The ICC ranged between 0.833 and 0.964 for inter-examiner agreement and between 0.717 and 0.979 for intra-examiner agreement. The ICC and Cronbach’s alpha for CPQ_11 − 14_ were 0.830 and 0.812, respectively.

### Data analysis

Descriptive statistical analysis was conducted using IBM SPSS Statistics for Windows, version 21 (IBM Corp., Armonk, NY, USA). Confirmatory factor analysis and structural equation modelling were carried out using Stata software, version 22.0 (Stata Corp., College Station, USA). Sociodemographic variables were initially described for the baseline sample, participants who completed the one-year follow up and those who were lost during the follow up. Pearson’s chi-square test and Mann-Whitney test were used to compare categorical and continuous variables between the analytic sample and participants lost during one-year follow up. Participants were distributed into four sociodental needs groups as follows. The “No dental conditions group” included adolescents without any of the oral conditions assessed. “BMD group” was composed of those without impact-related need and presenting malocclusion, at least one tooth with dental trauma and/or at least one tooth with dental calculus. “BMD with impact group” were adolescents with malocclusion, dental trauma and/or dental calculus, and impact-related need. “DNLP group” included participants with at least one decayed tooth and/or at least one tooth with clinical consequences of untreated dental caries.

Descriptive analysis reported the distribution of the study variables for the total sample and according to sociodental needs groups using means and standard deviations for continuous variables and proportions for categorical variables. Variables were compared between sociodental need groups using Pearson’s chi-square test and Kruskal-Wallis test for categorical and continuous variables, respectively. The proportion of adolescents with NN and propensity-related need were compared using the McNemar test. Moreover, the proportion of participants with NN for each dental condition and the respective impact-related need were compared using the McNemar test.

The measurement model involving socioeconomic status, OHRQoL at baseline and OHRQoL at one-year follow up latent variables and associated indicators was tested using confirmatory factor analysis. The indicators of socioeconomic status were monthly family income, house crowding and number of goods. OHRQoL indicators were the scores of the CPQ_11 − 14_ dimensions: symptoms, function, emotional and social.

Structural equation modelling using Maximum likelihood estimation method tested the proposed conceptual theoretical model and assessed the direct and indirect relationships between observed and latent variables (Fig. 1). The observed variables were sociodental needs (1 = no dental conditions, 2 = BMD, 3 = BMD with impact, 4 = DNLP group), propensity-related need (1 = low, 2 = moderate, 3 = high), use of dental services at one-year follow up (1 = no, 2 = yes), incidence of dental caries and incidence of filled teeth after one year. First, model identification and fit indices of the full model were evaluated and adjusted. Second, non-significant paths were removed from the full model which was re-estimated to obtain a statistically parsimonious model. Standardized root-mean- square residual (SRMR) ≤ 0.08 and comparative fit index (CFI) ≥ 0.90 were employed to assess the adequacy of the measurement, full and parsimonious models [20]. The significance level established for all analyses was 5% (*P* ≤ 0.05).

### Ethical aspects

This research was conducted in accordance with the Declaration of Helsinki and approved by the Research Ethics Committee of the Federal University of Amazonas (Protocol No. 57273316.1.0000.5020). All parents signed a written informed consent form agreeing to their participation and that of their children in the study prior to data collection.

## Results

The demographic and socioeconomic variables did not differ statistically between participants who completed the follow up and those lost during the follow up (Table 1). The one-year follow up data collection was completed by 334 adolescents. The distribution of sociodemographic data, behavioural propensity and oral health measures for the total sample and according to sociodental needs groups are presented in Table 2. Most of adolescents were females (56.6%), from families with monthly income between half and one BMW (40.1%) and had low behavioural propensity (58.1%). Use of dental services in the follow up was reported by 57.4% of the participants. The average incidence of dental caries and filled teeth were 1.38 and 0.39, respectively. Statistically significant differences were found for sociodental needs groups with respect to decayed teeth and OHRQoL at baseline. More adolescents reported dental visit at one-year follow up in the DNLP group. Incidence of dental caries and incidence of filled teeth were significantly higher in the DNLP group. Individuals in the BMD with impact group had worse OHRQoL at one-year follow up (Table 2).

**Table 1:** Sociodemographic variables between participants who completed the one year and those lost during the follow-up

Variables	Participants at baseline (N = 415)	Participants at one-year follow up (N = 334)	Participants lost during one-year follow up (N = 81)	*P-Value*
*Baseline*				
Sex, n (%)				0.297^a^
Male	175 (42.2)	145 (43.4)	30 (37.0)	
Female	240 (57.8)	189 (56.6)	51 (63.0)	
Monthly family income, n (%)				0.709^a^
≤ ½ BMW	113 (27.2)	91 (27.3)	22 (27.2)	
> ½ – 1 BMW	163 (39.3)	134 (40.1)	29 (35.8)	
> 1 BMW	139 (33.5)	109 (32.6)	30 (37.0)	
House crowding, mean (SD)	1.58 (0.94)	1.59 (0.92)	1.50 (1.02)	0.173^b^
Number of goods, mean (SD)	6.70 (2.58)	6.66 (2.50)	6.86 (2.89)	0.630^b^

*P* values refer Pearson’s chi-square test^a^ and Mann-Whitney test^b^

**Table 2:** Sociodental needs groups according to sex, socioeconomic status, use of dental services, dental caries, restored teeth and OHRQoL

Variables	Total	No dental conditions	BMD	BMD with impact	DNLP	*P-value*
*Baseline*						
Sex, n (%)						0.580^a^
Male	145 (43.4)	5 (33.3)	42 (46.7)	35 (38.9)	63 (45.3)	
Female	189 (56.6)	10 (66.7)	48 (53.3)	55 (61.1)	76 (54.7)	
Monthly family income, n (%)						0.578^a^
≤ ½ BMW	91 (27.3)	3 (20.0)	27 (30.0)	23 (25.8)	38 (27.2)	
> ½ – 1 BMW	134 (40.1)	8 (53.3)	37 (41.1)	30 (33.7)	59 (42.1)	
> 1 BMW	109 (32.6)	4 (26.7)	26 (28.9)	36 (40.4)	43 (30.7)	
House crowding, mean (SD)	1.59 (0.92)	1.95 (1.47)	1.53 (0.90)	1.57 (0.86)	1.62 (0.89)	0.698^b^
Number of goods, mean (SD)	6.66 (2.50)	6.27 (2.63)	6.27 (2.52)	7.13 (2.67)	6.66 (2.32)	0.134^b^
Behavioural propensity, n (%)						0.458^a^
Low	194 (58.1)	7 (46.7)	49 (54.4)	56 (62.2)	82 (59.0)	
Moderate	76 (22.8)	3 (20.0)	25 (27.8)	21 (23.3)	27 (19.4)	
High	64 (19.2)	5 (33.3)	16 (17.8)	13 (14.4)	30 (21.6)	
Decayed teeth, mean (SD)	0.88 (1.54)	0.00 (0.00)	0.00 (0.00)	0.00 (0.00)	2.12 (1.74)	< 0.001^b^
Filled teeth, mean (SD)	0.51 (1.04)	0.47 (0.74)	0.53 (1.08)	0.53 (1.11)	0.50 (1.00)	0.937^b^
OHRQoL, mean (SD)	14.42 (8.90)	10.80 (7.50)	7.70 (4.70)	19.16 (8.92)	16.10 (8.45)	< 0.001^b^
*One-year follow up*						
Use of dental services, n (%)						0.004^a^
Yes	192 (57.4)	6 (40.0)	45 (50.0)	45 (50.0)	95 (68.8)	
No	142 (42.6)	9 (60.0)	45 (50.0)	45 (50.0)	43 (31.2)	
Dental caries incidence, mean (SD)	1.38 (2.39)	0.93 (1.49)	1.19 (2.26)	1.26 (2.33)	1.64 (2.58)	< 0.001^b^
Filled teeth incidence, mean (SD)	0.39 (0.94)	0.00 (0.00)	0.17 (0.46)	0.24 (0.64)	0.68 (1.26)	< 0.001^b^
OHRQoL, mean (SD)	13.95 (8.63)	13.33 (10.44)	10.19 (6.60)	16.52 (9.44)	14.78 (8.30)	< 0.001^b^

BMD: Basic model of dental needs in children (≥ 1 tooth with dental calculus, ≥ 1 tooth with dental trauma without pulp exposure and/or malocclusion)

DNLP: dental needs for life-threatening and progressive oral conditions (≥ 1 decayed tooth and/or ≥ 1 tooth with clinical consequences of untreated dental caries)

Oral impact: ≥ 1 item with CPQ_11 − 14_ score “often” or “very often”

*P-*values refer Pearson’s chi-square test^a^ and Kruskal-Wallis test^b^

An additional file presents the distribution of participants according to NN, impact-related need, and propensity-related need [see Additional file 1]. The proportion of adolescents with a NN for dental treatment was 95.5%, of which 43.6% had dental needs life-threatening and progressive oral conditions (DNLP). Of these adolescents, 9.4% had high behavioural propensity and would be able to undergo immediate dental treatment. The remaining 34.2% with low or medium behavioural propensity could receive education and/or oral health promotion (DHE/OHP) along with clinical treatment. Of the 56.4% adolescents within the basic model of dental needs (BMDN), 28.2% did not have oral impacts and 28.2% had their quality of life affected by oral conditions. Of these, 4.1% had high behavioural propensity-related need and should be treated as initially planned. The remaining 24.1% adolescents with BMD and oral impacts had medium or low behavioural propensity and would need DHE/OHP [see Additional file 1]. The proportion of adolescents with NN (95.5%) was statistically different from those classified as high-level of behavioural propensity (13.5%) (*P* < 0.001, McNemar test). In addition, the proportion of adolescents with NN according to each dental condition was significantly different than the respective impact-related need. In total, NN estimates were higher than impact-related need for dental caries (37.4% vs. 21.0%, *P* < 0.001), clinical consequences of untreated dental caries (19.5% vs. 12.9%), malocclusion (85.0% vs. 43.7%, *P* < 0.001), dental trauma (17.4% vs. 8.1%, *P* < 0.001) and dental calculus (58.7% vs. 33.5%, *P* < 0.001).

**Fig. 2:**
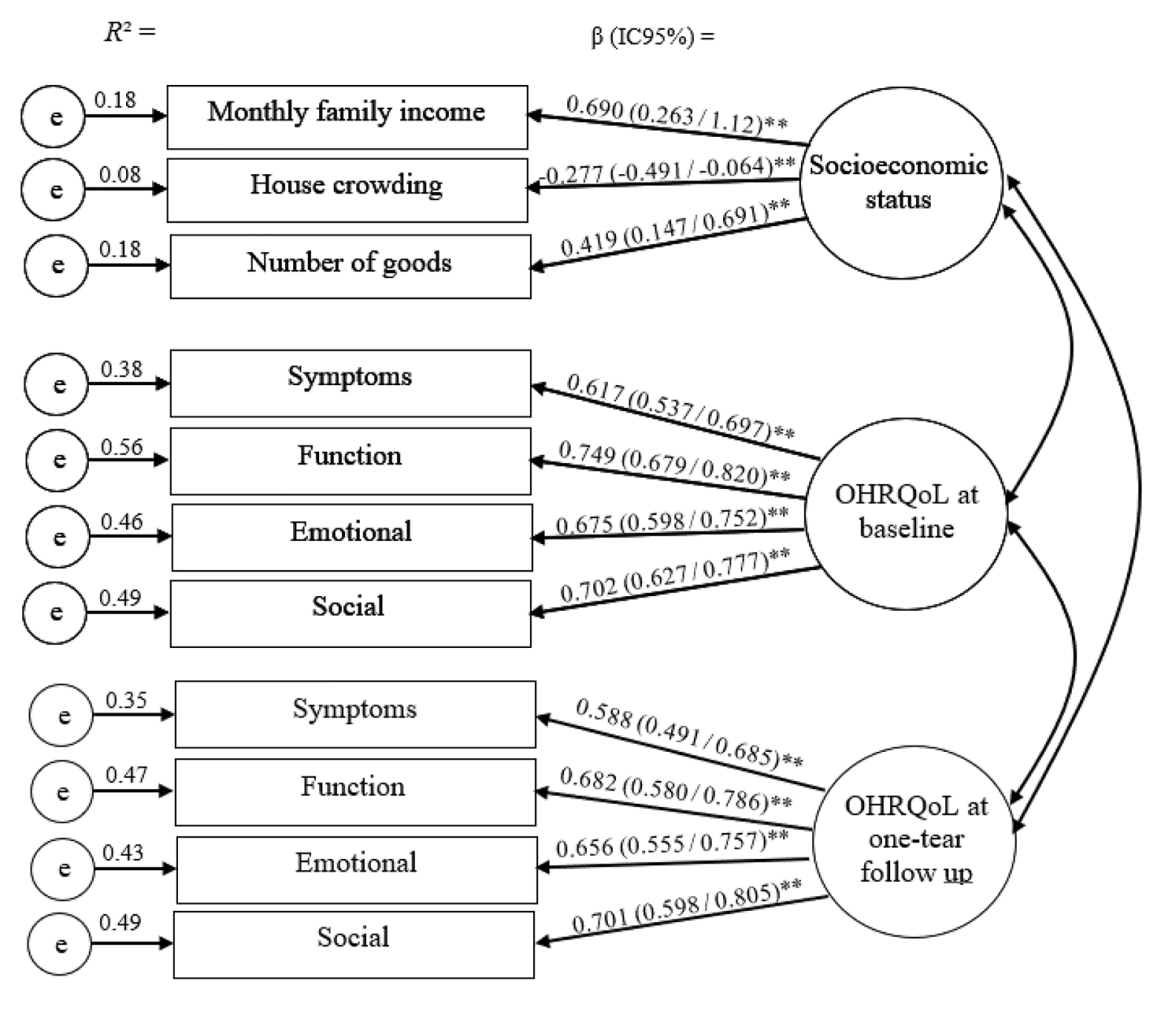
Confirmatory factor analysis of the 3-factors 11 items (measurement model) β: standardized coefficients (95% confidence intervals) **Significant standardized coefficients (*P* < 0.01)

The measurement model was assessed using confirmatory factor analysis for the latent variables socioeconomic status, OHRQoL at baseline and OHRQoL at one-year follow up. The items that confirmed the latent variable socioeconomic status were monthly family income (β = 0.690), house crowding (β= -0.277) and number of goods (β = 0.419). The item loadings confirming the latent variable OHRQoL at baseline were symptoms (β = 0.617), function (β = 0.749), emotional (β = 0.675) and social (β = 0.702) and OHRQoL at one-year follow up were symptoms (β = 0.588), function (β = 0.682), emotional (β = 0.656) and social (β = 0.701) (Fig. 2). The fit indices for the measurement model were SRMR = 0.051 and CFI = 0.918.

Structural equation modeling supported the hypothetical full model with the following values: SRMR = 0.077 and CFI = 1.000. Non-significant direct relationships between variables were removed and the parsimonious model was estimated reaching adequate fit indices (SRMR = 0.054 and CFI = 0.935).

**Fig. 3:**
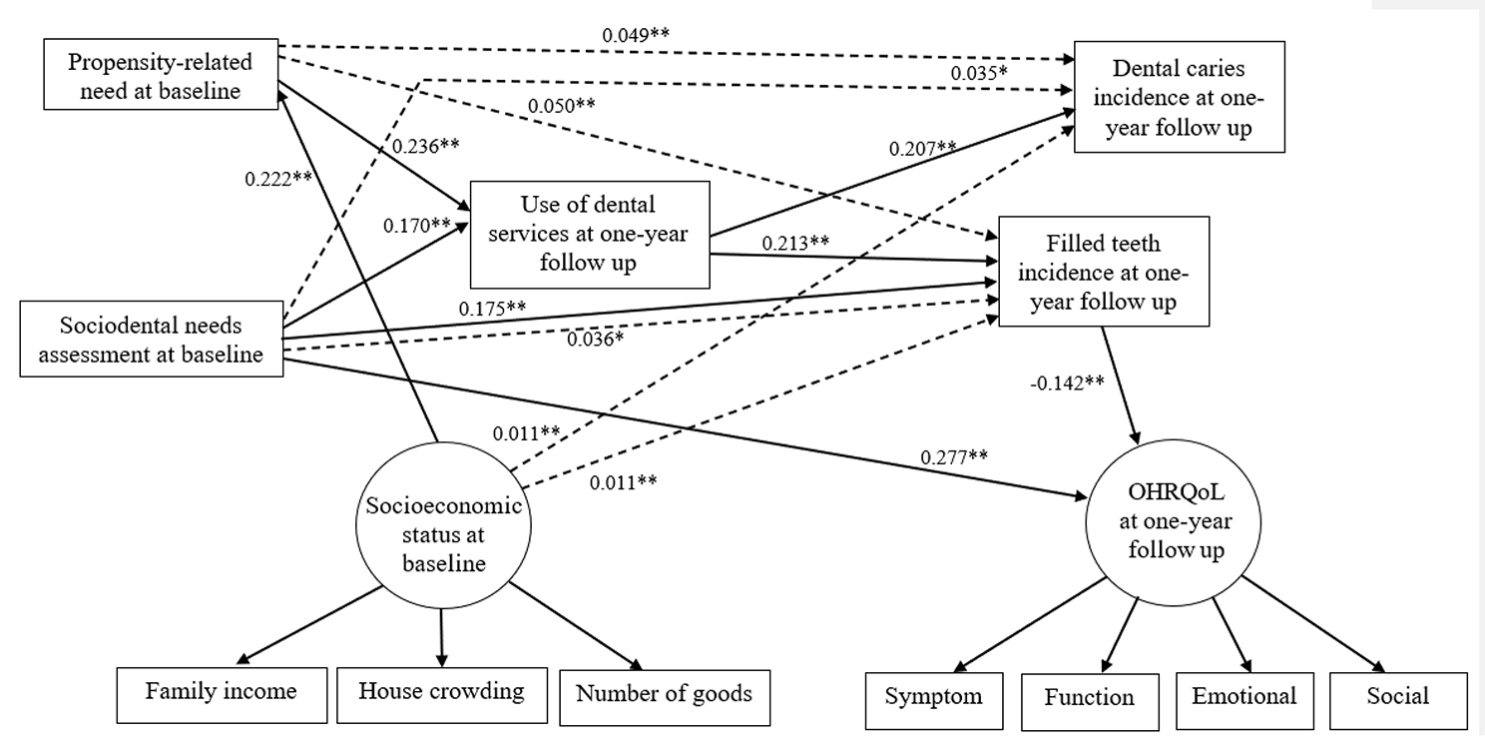
Parsimonious model of associations between sociodental needs measures, socioeconomic status, use of dental services, dental caries, filled teeth, and oral health-related quality of life Sociodental needs assessment at baseline: normative need and impact-related need at baseline Solid lines indicate standardized direct effects and dashed lines indicate standardized indirect effects * *P* < 0.05; ** *P* < 0.01

The direct and indirect relationships estimated in the parsimonious model are summarized in Fig. 3. Sociodental needs directly predicted greater use of dental services (β = 0.170), higher incidence of filled teeth (β = 0.175) and worse quality of life at one-year follow up (β = 0.277). Higher behavioural propensity was directly associated with greater use of dental services at one-year follow up (β = 0.236). Incidence of dental caries (β = 0.207) and incidence of filled teeth (β = 0.213) were directly predicted by use of services. Higher incidence of filled teeth was related to better quality of life (β= -0.142). Greater socioeconomic status directly predicted better behavioural propensity (β = 0.222). Incidence of dental caries and incidence of filled teeth were indirectly predicted by sociodental needs and behavioural propensity-related needs via use of dental services. Socioeconomic status indirectly predicted incidence of dental caries and incidence of filled teeth via behavioural propensity-related needs and use of dental services (Fig. 3).

## Discussion

The present longitudinal study compared the dental treatment needs between normative need and the sociodental dental needs according to life-threatening and progressive oral conditions (DNLP) and basic model of dental needs (BMDN) in 12-year-old adolescents from families living in deprived communities in Brazil. In addition, the influence of sociodental system of dental needs assessment on use of dental services, dental clinical outcomes and OHRQoL over one year of follow up was also investigated.

The substantial decrease of the sociodental approach in the estimates of treatment needs from 95.5% (normative need) to 13.5% (high propensity-related need) is a confirmatory finding. Although almost half of the adolescents (43.6%) had DNLP and would require immediate dental treatment, nearly 10% had high behavioural propensity and thus would be able to efficiently benefit from treatments. These findings are consistent with previous studies that suggested that normative methods of needs assessment overestimate the proportion of subjects in need of dental treatment than the sociodental approach [2, 6–11]. However, evidence on the role of the sociodental system of dental needs assessment on use of dental services and oral health outcomes over time is scarce.

The present findings partially confirmed the influence of sociodental needs approach on use of dental services and oral health measures of adolescents living in deprived communities. Adolescents with greater treatment needs according to normative need and impact-related need and those with higher behavioural propensity at baseline were more likely to use dental services and receive more restorative treatments over one-year period. Therefore, our findings support the importance of incorporating subjective and social measures in the assessment of oral health needs since the sociodental approach predicted dental visits and treatment for dental caries. Use of dental services can be influenced by demographic and socioeconomic factors related to general and oral health [21, 22]. The findings of the present study also showed that use of dental services expressed adolescent’s oral health needs when normative measures and oral impacts were considered. However, socioeconomic status was not associated with use of dental services. Studies analyzing the above-mentioned relationships over time are scarce and report controversial results about the direction of these relationships [23].

Use of dental services was a meaningful variable in this study since greater sociodental needs and higher behavioural propensity were associated with higher incidence of dental caries and filled teeth mediated by use of dental services. The possible positive influence of use of dental services on OHRQoL was not observed in the present study. So, the potential benefits of dental visits on preventing dental caries and improving OHRQoL were not confirmed. The use of dental services failed to prevent dental caries, suggesting that attending dental visits are not sufficient to reduce the development of new dental caries lesions in adolescents who are also at greater risk of future caries [24]. A recent cohort study demonstrated that the type of dental care received can influence oral health outcomes as dental caries increase was more common among children who underwent curative treatment [25]. Thus, disease-centred dental care focusing on curative interventions possibly predisposes the individuals to a dental restorative cycle. This model of dental care fails to recognize the contemporary understanding of dental caries pathogenesis and is inadequate to tackle the global burden of oral diseases [26]. There is no consensus on the impact of dental treatments and frequency of use of dental services on quality of life of children and adolescents [27, 28]. The observational nature of this study might explain such findings since the types of dental treatments received by the participants during the one-year follow up was not registered.

Behavioural propensity was directly related to use of dental services and indirectly associated with incidence of filled teeth. The association between oral health behaviours and use of dental services was reported by another study that emphasized the importance of adopting healthy behaviours among children and adolescents as they can determine the pattern of oral diseases in the near future [29]. In the present study, the association between better socioeconomic status and higher behavioural propensity is supported by previous evidence, reinforcing the predictive role of socioeconomic status in the adoption of health behaviours, and occurrence of risk factors and diseases [30].

The use of structural equation modelling was a robust analytical method to test the hypotheses of theoretical model, and to explore the simultaneous relationships between the variables related to the sociodental system of dental needs assessment, socioeconomic status, use of dental services, dental caries and OHRQoL over one year of follow up. Furthermore, the longitudinal design of this study supports the interpretation of the temporal relationships between variables. Nonetheless, some limitations must be acknowledged. First, only 12-year-old adolescents living in social deprivation were included. So, the generalization of our findings to other age groups and adolescents from different socioeconomic backgrounds must be carefully proceeded. Second, oral health behaviours were assessed only at baseline and some participants may have changed the investigated behaviours during the study period. Third, other potential predictors of dental services utilization, dental caries and OHRQoL, such as parental practices and psychosocial factors, were not analyzed in this study. Finally, relevant information related to use of dental services at one-year follow up, such as the reason for dental visit and the types of dental treatments received during the follow up were not assessed.

## Conclusion

The longitudinal evaluation of the sociodental approach on use of dental services, dental caries incidence and OHRQoL demonstrated that normative need combined with impact-related need and propensity-related need can provided a sound strategy for oral health treatment planning. Our findings also suggest that use of dental services was a relevant factor by which the sociodental needs and behavioural propensity impact on oral health afterwards. Access only to dental care in deprived communities resulted in more filled teeth but did not prevent future lesions of dental caries and did not improve OHRQoL.

Future interventional studies are necessary to evaluate the acceptability of the sociodental approach for delivering oral health care as well as the benefits of the sociodental system to improve adolescents’ dental clinical measures and subjective oral health. Investigations on the impact of oral health education and/or health promotion programs on behavioural propensity and oral health outcomes are also needed.

## Electronic supplementary material

Below is the link to the electronic supplementary material.

Supplementary Material 1

## Data Availability

The data that support the findings of this study are available from the Dental School, Federal University of Amazonas but restrictions apply to the availability of these data, which were used under license for the current study, and so are not publicly available. Data are however available from the author Profa Maria Augusta Bessa Rebelo (email: augusta@ufam.edu.br) upon reasonable request and with permission of the Dental School, Federal University of Amazonas.

## Abbreviations

BMDN: Basic model of dental needs
CFA: Confirmatory factor analysis
DMFT: Decayed, Missing and Filled Teeth Index
DNLP: Dental needs for life-threatening and progressive oral conditions
IRN: Impact-related need
NN: Normative need
OHRQoL: Oral health-related quality of life
PUFA/pufa index: Pulpal involvement (P/p), ulceration caused by dislocated tooth fragments (U/u), fistula (F/f) and abscess (A/a)
SEM: Structural equation modeling
