# Supplementary material for: Does using the sociodental approach in oral health care influence use of dental services and oral health of adolescents living in deprived communities? a one-year follow up study
Source: BMC Health Serv Res. 2023 Jun 9;23:605. doi: 10.1186/s12913-023-09596-0 (PMC10251576; doi:10.1186/s12913-023-09596-0)
Supplement: Supplementary file 1 — Supplementary Material 1 [file 12913_2023_9596_MOESM1_ESM.docx]

Additional File 1. Social dental needs of 334 adolescents.

95.5% (N = 319)

Normative need

*No*

*Yes*

*Emergency/progressive conditions*

Emergency/progressive conditions

**DNLP**

**BMDN**

**56.4 (N=180)**

**43.6 (N=139)**

*Yes*

*Oral impacts on quality of life*

*No*

**28.2 (N=90)**

Impact-related need

*Behavioural propensity*

**9.4 (N=30)**

**4.1 (N=13)**

**Propensity-related need**

High propensity:

Initially planned treatment

**34.2 (N=109)**

**24.1 (N=77)**

Medium/low propensity:

Most appropriate treatment + DHE/OHP

**28.2 (N = 90**

DHE/OHP

Total DHE/OHP

86.5% (N = 276)

DHE/OHP: dental health education and/or oral health promotion

BMD: Basic model of dental needs in children

DNLP: dental needs for life-threatening and progressive oral conditions
